# Antimicrobial Resistance Along the Food Chain: Spread and Integrated Strategies for Mitigation and Control

**DOI:** 10.3390/antibiotics15030311

**Published:** 2026-03-19

**Authors:** Anna Maria Spagnolo, Francesco Palma, Giulia Amagliani, Michele Fernando Panunzio, Maria Teresa Montagna, Elena Alonzo, Guglielmo Bonaccorsi, Giulia Cairella, Emilia Guberti, Giuditta Fiorella Schiavano

**Affiliations:** 1 Department of Health Sciences, University of Genova, Via Pastore 1, 16132 Genova, Italy; 2 Department of Biomolecular Sciences, University of Urbino Carlo Bo, 61029 Urbino, Italy; 3 Department of Prevention, Food Hygiene and Nutrition Service, ASL Foggia, 71121 Foggia, Italy; 4 Interdisciplinary Department of Medicine, Hygiene Section, University of Bari Aldo Moro, 70124 Bari, Italy; 5 Servizio Igiene degli Alimenti e Nutrizione (SIAN), Azienda Sanitaria Provinciale, 95100 Catania, Italy; 6 Department of Health Science, University of Florence, Viale GB Morgagni 48, 50134 Florence, Italy; 7 Department of Prevention, Unit Local Health Roma 2, 00159 Rome, Italy; 8 Società Italiana Igiene, Medicina Preventiva e Sanità Pubblica, 00144 Rome, Italy; 9 Department of Humanities, University of Urbino Carlo Bo, 61029 Urbino, Italy

**Keywords:** food, antimicrobial resistance, One Health, antimicrobial stewardship, surveillance

## Abstract

The development of antimicrobial resistance (AMR) and the emergence of multiresistant pathogens represent a growing global threat to both human and animal health. Beyond the excessive and improper use of antimicrobials in human medicine, irrational use in veterinary medicine, agriculture, and aquaculture significantly contributes to the selection and spread of resistant microorganisms, which can enter the food chain and reach humans through food consumption or handling. Based on results from a recent meta-analysis, the prevalence of antimicrobial-resistant foodborne pathogens in food samples exceeds 10%. The veterinary sector is of particular concern, as a large proportion of antimicrobials are used in animal production, generating strong selective pressure and favoring the dissemination of AMR along the food chain. In an increasingly interconnected global context, resistant pathogens and resistance determinants can disseminate rapidly across sectors and national borders, making strategies confined to a single sector insufficient; therefore, effectively addressing AMR requires a One Health approach encompassing the human, veterinary, and environmental domains. Key mitigation strategies include strengthening antimicrobial stewardship programs, also in animal production, reducing routine prophylactic use of antimicrobials, and improving surveillance, coordinated across sectors and, where possible, further supported by advanced technologies such as artificial intelligence and machine learning. Further efforts are also needed to improve microbiological diagnostics, particularly through rapid and molecular methods, to support timely, targeted therapies and reduce inappropriate empirical treatments. In parallel, investment in new therapeutic options, including innovative molecules, drug combinations, and alternative approaches, remains crucial to effectively countering the growing burden of antimicrobial resistance.

## 1. Introduction

Antimicrobial resistance (AMR) is a serious global public health problem and a threat to modern medicine, with both clinical (increased mortality and longer hospital stays) and economic impacts [1,2,3].

In 2021, approximately 4.71 million (95% UI 4.23–5.19) deaths were associated with bacterial AMR, of which 1.14 million were attributable to it [1]. If the trend persists, it has been estimated that the burden of AMR is expected to increase to 8.22 million (6.85–9.65) associated deaths and 1.91 million (1.56–2.26) attributable deaths in 2050 [4].

The inappropriate use of antimicrobials is the main factor responsible for the development of drug-resistant pathogens.

In addition to the excessive and improper use of antimicrobials in human medicine, irrational use in veterinary medicine and agriculture contributes significantly to the development and further spread of resistant pathogens, which can then enter the food chain and reach humans through food consumption or handling.

Based on results from a recent meta-analysis of 332 studies conducted across 36 countries between 2010 and 2020, the prevalence of antimicrobial-resistant foodborne pathogens in food samples exceeds 10%. The analysis also showed that resistance was most frequently observed against β-lactam antibiotics, with *Bacillus cereus* exhibiting the highest resistance rate (94%) [5].

This review aims to provide a comprehensive overview of the emergence and spread of AMR along the food chain, with a focus on antibiotic use in food production systems, major foodborne-resistant pathogens, underlying molecular mechanisms, and the public health implications of AMR. Furthermore, current challenges and integrated mitigation strategies within a One Health framework are discussed, highlighting the role of antimicrobial stewardship, surveillance, and innovation in diagnostics and therapeutics.

A core feature of this review is the integration of evidence from different sectors of the food chain together with relevant regulatory frameworks, providing a broader perspective on the emergence and spread and control of antimicrobial resistance in the food system.

## 2. Use of Antibiotics in Food Production and Transmission in Food

### 2.1. Use of Antibiotics in Farms and Transfer of AMR Strains in Animal By-Products

Antimicrobial resistance poses an urgent threat to global public health, in part driven by the intensive and irrational use of antibiotics in animal farming. It is estimated that over 70% of antimicrobials produced worldwide are used in food–animal production [6]. Moreover, it must be considered that the widespread use of antibiotics in medicine and veterinary medicine has led to the selection of multi-antibiotic-resistant bacteria [7].

In animal farming, the use of antibiotics can be divided into three main categories:

(1) Antibiotic Growth Promoters (AGPs): Antibiotics are administered at low, sub-therapeutic doses continuously in feed or water, with the primary aim of maximizing growth yield or improving feed conversion efficiency. This practice has been recognized as the main cause of resistance selection, due to long-term exposure to low doses, which favor the emergence of resistant strains. In this regard, the European Union (EU) banned the use of AGPs in 2006 [8]. Despite the ban in the EU, the regulation of AGPs is still poor in many parts of the world [6]. In the United States, the use of medically important antibiotics for growth promotion has been restricted and placed under veterinary supervision through the Veterinary Feed Directive (VFD), which came into full effect in 2017 [9].
(2) Therapeutic: Antimicrobials are administered to individual clinically ill animals to treat infections. Although necessary for animal health and well-being, they must be guided by the principles of prudent use under veterinary supervision.
(3) Prophylactic/Metaphylaxis: Drugs are administered in specific situations or to individual animals to reduce the risk of infection (e.g., after surgery) or to an entire group of animals (mass medication) to reduce the incidence or severity of disease (e.g., when part of the group is already infected) [10]. It should be noted that this mass approach poses a high risk of AMR selection, especially in high-density farms. Modern intensive farming systems represent a major driver for antimicrobial use, creating conditions that support the rapid spread of infections.

In these environments, routine antibiotic use is often necessary to compensate for the high risk of disease, especially if biosecurity, management, and vaccination measures are not enough. In addition, increasing numbers of nations, particularly low- and middle-income countries (LMICs), are adopting these intensive systems due to growing global demand for animal protein. This intensification is expected to lead to a 67% increase in global antimicrobial use by 2030 [6]. However, this projection is based on modeled scenarios and is subject to uncertainty, with regional variability depending on livestock production trends, economic development, and the implementation of antimicrobial stewardship policies.

The spread of AMR from animals to humans and the environment is a ‘One Health’ issue that recognizes the interconnectedness of human, animal, and environmental health. The use of antibiotics in intensive animal farming can lead to the contamination of air, soil, and water with drug residues and resistant bacteria (ARBs). Animal manure and slurry, when used as fertilizer without proper treatment, can transfer resistance genes (ARGs) and ARBs to soil, water, and even crops, completing an environmental cycle of resistance. Moreover, intensive animal farming releases high concentrations of bioaerosols into the environment, mainly composed of microorganisms, including ARBs [7]. In recent years, considerable attention has been paid to animal by-products, which can become vehicles for the transmission of ARBs. Derived products such as meat, milk, and eggs may come into contact with ARBs present in the animal, so care must be taken to identify the critical points at which contamination may occur.

AMR can be transmitted to humans through the consumption or handling of contaminated food of animal origin, such as meat and meat products. Relevant microorganisms under surveillance include *Salmonella* spp., *Campylobacter* spp., *Escherichia coli* (resistance indicator), and methicillin-resistant *Staphylococcus aureus* (MRSA). Another important step in the EU to combat antimicrobial resistance has been the introduction of Regulation (EU) 2019/6, through which a ban on the use of antibiotics as growth promoters has been confirmed, severely restricting their prophylactic use, and allowing it only in exceptional cases on individual animals or small groups, and where there is a very high risk of infection [11]. The regulation also established the principle of prudent and responsible use of antimicrobials, laid the foundations for the mandatory collection of data on the sale and use of antibiotics in farm animals, and provided for the possibility of reserving certain classes of antimicrobials exclusively for human medicine. On this basis, Implementing Regulation (EU) 2022/1255 gave concrete effect to the provisions of the framework regulation by identifying a list of antimicrobials reserved exclusively for human use [12]. A further step forward is represented by Implementing Regulation (EU) 2024/1973, which will be applicable from 8 August 2026 [13]. This act regulates in more detail the exceptional (‘cascade’) use of antimicrobials, i.e., cases where drugs not specifically authorized for a given species or indication are used, establishing that when such exceptional use is made, the choice of antibiotic must be based, where possible, on the identification of the pathogen and antimicrobial susceptibility testing.

It must be emphasized that contamination is not limited to ARBs but also includes residues of antibiotics used. For example, residues of tetracyclines, β-lactams, and cephalosporins have been quantified in chicken meat, bovine carcasses, rabbit meat/liver/kidneys, beef, milk, and eggs in African countries, often exceeding the levels recommended by the World Health Organization (WHO) [14]. The presence of drug residues poses risks to public health, including allergic reactions (often associated with β-lactams), genotoxic or carcinogenic effects (often related to chloramphenicol, sulfamethazine, nitrofurans), and promoting the development of ARBs [15].

### 2.2. Use of Antibiotics in Aquaculture: AMR in Fish and Shellfish Products

Aquaculture is a rapidly expanding food production sector, driven by growing global demand for healthy protein sources. The transition from semi-intensive to intensive farming systems has led to an increase in aquaculture production, but the use of subtherapeutic antibiotic levels to control disease within these farms has turned aquatic ecosystems into “AMR hotspots” [16]. It should be noted that approximately 75% of antibiotics administered are not completely absorbed or digested by fish and are released into the surrounding environment through feces and urine, while 50% of their not degraded metabolites have been detected in fishponds, thus promoting the development of ARBs [17]. It is also important to consider the potential transmission of AMR from bathing waters to bathers who spend a lot of time in contact with these contaminated waters. Leonard et al. demonstrated that prolonged contact with coastal waters contaminated by *bla*_CTX-M_-bearing *E. coli* tripled the resistance of surfers’ microbiome to β-lactams [18].

Zhang and colleagues [19] reported that around 24% of resistance genes pose a threat to both human and animal health. Multi-drug-resistant bacterial strains are frequently detected in fish, shellfish, and the aquatic environment. Among the most frequently isolated resistant bacteria are *Vibrio* spp., *Aeromonas* spp., *Bacillus* spp., *Pseudomonas* spp., *Flavobacterium*, and Enterobacterales [16,20]. Some of the most frequently detected antibiotic resistance genes, along with their respective antibiotic classes, are tetracycline (*tetA*, *tetB*, *tetK*, *tetM*), quinolone (*qnrA*, *qnrB*, *qnrS*), and sulfonamides (*sulI*) [16].

Gabucci et al. demonstrated that *Arcobacter butzleri* strains, isolated from sushi and other matrices, were multi-drug-resistant (MDR), with 100% resistance to tetracyclines and cefotaxime [21].

As with animal farming, the use of antibiotics in aquaculture is highly regulated, and increasing numbers of countries are imposing maximum residue levels (MRLs) of antibiotics in fish muscle or skin. The EU imposes an MRL of 100 µg/kg for fluoroquinolones and sulfonamides, while the MRLs for penicillins are 4 µg/kg [16,22]. The Food and Drug Administration (FDA) approved only oxytetracycline, a combination of sulfadimethoxine and ormetoprim, and florfenicol for specific fish species and diseases [23]. In China, an MRL of 100 ng/g has been established for enrofloxacin, ciprofloxacin, and total sulfonamides, while for chloramphenicol and furazolidone, the limit is 0 ng/g [22].

From 2012 to 2021, the Experimental Zooprophylactic Institute of Umbria and Marche (Central Italy) performed an antibiotic residue monitoring in animal products [24]. A total of 2354 samples from 444 farms in the Umbria and Marche regions were collected. The food matrices examined included animal muscle (beef, pork, poultry, turkey, and fish), cow’s milk, and chicken eggs. Among the fish muscle samples analyzed, none were found to be non-compliant with the MRLs established by EU Regulation 37/2010 [25]. Only one sample was positive for flumequine residues in fish muscle; however, this was still classified as compliant with current regulations, as the detected concentration was 20.0 µg/kg.

A crucial risk to Public Health is that for many commonly used antibiotics (such as oxytetracycline, tetracycline, and enrofloxacin), the Minimum Selective Concentration is lower than the respective MRLs established by EU Regulation 37/2010 [24,25]. This suggests that the concentrations legally permitted in fish products considered “compliant” could still contribute to the spread of AMR. Current MRLs are primarily established based on Acceptable Daily Intake (ADI) values and consumer safety assessments, which focus on direct toxicological effects. However, sub-therapeutic concentrations of antimicrobials, even when compliant with existing regulations, may still exert selective pressure on microbial communities, potentially contributing to the emergence and dissemination of AMR. Therefore, it may be appropriate to integrate AMR considerations into future risk assessment and policy development.

In summary, the accumulation of antibiotics and the prevalence of ARBs in aquaculture not only threaten the functioning of aquatic ecosystems but also pose a potential risk to human health through the food chain.

### 2.3. Presence of Antibiotic Residues and AMR in Horticulture and Agriculture

As reported above, a large percentage of antibiotics produced are used in animal farming, particularly in intensive farming. Since animals poorly assimilate these drugs, it is estimated that approximately 25–75% of antibiotics are excreted as active parent compounds or metabolites into the surrounding environment [26]. These compounds can contaminate agricultural soils through the application of animal manure, sewage sludge, or irrigation with reclaimed wastewater. Zhao and colleagues reported that a concentration up to 225.45 mg kg^−1^ and 1420.76 mg kg^−1^ of norfloxacin and enrofloxacin, respectively, can be found in chicken manure [27]. Furthermore, Huygens and colleagues detected residues of doxycycline, oxytetracycline, ciprofloxacin, enrofloxacin, flumequine, and lincomycin in 100% of fattening calf slurry samples analyzed [28]. The use of manure as fertilizer in the agricultural field is one of the ways that antibiotic residues can contaminate the environment, contributing to the spread of ARBs. To our knowledge, regulatory MRLs for veterinary antibiotics are established only for edible animal tissues and products (e.g., muscle, liver, kidney, fat, milk, and eggs) within the framework of food safety regulations, while no specific regulatory thresholds are defined for antibiotic residues in manure.

Antibiotics present in the soil can be absorbed by plant roots and transferred along the food chain [26]. A study has shown that adding 20 mg kg^−1^ chlortetracycline hydrochloride to the soil, antibiotic accumulation in lettuce roots reached 1.16 mg kg^−1^ [29]. Sulfamethoxazole was also detected in high concentrations in eggplant and bell pepper fruits, correlating with its concentration in the soil [30]. The measurement of such residues is typically performed using sensitive techniques such as high-performance liquid chromatography coupled with tandem mass spectrometry (HPLC-MS/MS) [31]. It should be noted that in agriculture, many kinds of antibiotics are used to treat plant diseases. Table 1 shows some of the main antibiotics used in horticulture and agriculture.

In a study conducted by Abraham et al. on vegetables, they revealed alarming MDR, with isolates of *E. coli* and *Klebsiella pneumoniae* resistant to ≥3 classes of antibiotics, including critical resistance to gentamicin, due to the use of untreated manure [38]. In another study, carrot and spinach samples grown in soil amended with sewage sludge showed that ARGs and mobile genetic elements (MGEs) were two to four times more abundant than in the controls [39]. In particular, the *intl1* and *IS26*, *bla*_CTX_ and *qnrS* genes were higher in the treated plant samples. For spinach, the abundance of ARGs was higher in shoot tissues (leaves) than in roots. In light of these, it must be considered that the presence of antibiotics and ARGs in vegetables can transfer MDR to the human digestive tract microbiome following consumption [39]. Analysis of strains isolated from fresh fruit and vegetables revealed that 74% of isolates were resistant to at least one antibiotic, and 16% (14 out of 88) were classified as MDR [40]. It should be emphasized that the use of antibiotics on cropland can have long-term ecological implications, as soils can serve as reservoirs of ARGs that spread through microbial communities and potentially alter soil ecosystem functioning, affecting key processes such as organic matter decomposition, nitrification, denitrification, and carbon and nitrogen cycles [41]. Therefore, great care must be taken when using antibiotics in crops to reduce the risk of developing ARBs.

### 2.4. Environmental Contamination and Cross-Contamination During Processing and Distribution

The introduction of raw materials (meat, fish, vegetables) contaminated with antibiotic residues and/or ARBs into the food chain can increase the risk of cross-contamination. During the production chain, contamination can occur at any stage of the food chain, like the slaughter and processing phases of meat, which represent a crucial point in the transmission of ARGs and ARBs from farm to table [42]. Another critical point is the meat scalding stage, during which contamination could occur due to the use of water contaminated with antibiotic residues or ARBs. During this stage, *Salmonella* and *S. aureus* are common, and the abundance of MGEs such as *intI 1* is high [42]. Equally critical for cross-contamination is the evisceration stage, in which the removal of organs can cause the spread of ARGs (such as *sul2*) and ARBs, contaminating the carcass and subsequent stages. Cross-contamination is the main source of most AMR cases detected at this stage [42]. As reported in a systematic review by Wu et al., the cross-contamination was estimated at 29% up to 69% of the whole contaminated carcasses [43]. It should be noted that the wide variability in cross-contamination rates could be due to differences in study design, settings, and sampling approaches across the studies included in the systematic review. Moreover, in the same study, a difference in the cross-contamination rate between an HACCP (60.87%) and non-HACCP (98.48%) slaughterhouses was reported [43]. Also, the storage, transportation, sales, and food services are equally important. Ice used for storing meat and fish products can carry MDR microorganisms (e.g., *S. aureus* and *V. parahaemolyticus*), and in this phase, the contamination can result from dirty ice machines, unhygienic handling, or contaminated water [44].

The spread of AMR and the presence of MDR microorganisms are no longer limited to industrial environments, but are also emerging in domestic environments, in which food is handled daily. Azevedo and colleagues investigated the prevalence of AMR Enterobacterales in domestic food-related environments, and from this study emerged that 49.6% of the isolates were resistant to at least one antibiotic (32.8% to ampicillin, 6.4% to nitrofurantoin, 4% to tetracycline, 3.2% to nalidixic acid, 2.4% to chloramphenicol, and 1.7% to trimethoprim), while the 6.4% of the isolates resulted in MDR [45]. In this case, the bacteria were isolated from household objects, including door handles, refrigerators, dishwashers, stove knobs, taps, countertops, and kitchen towels. Cross-contamination in the domestic environment can take place at any moment, for example, when using the same cutting board or knife for raw meat and vegetables, using the same utensils for cooked and raw foods, or handling food with unwashed hands. It should be noted that the surface disinfection method can also impact the spread of AMR in these environments. Despite their high effectiveness, chemical disinfectants may leave biocide residues on surfaces that can promote the emergence and spread of antibiotic resistance in bacteria present in these environments, including pathogens [46]. This could be mainly due to the ability of some biocide residues to dissipate the membrane potential by affecting the transport of antibiotics with intracellular targets, as well as to their ability to overexpress some efflux pumps (e.g., *adeB* by benzalkonium and chlorhexidine) [47].

Moreover, it must be highlighted that people can easily transfer drug-resistant bacteria to food or equipment through inadequate food handling or poor hand hygiene [48]. For this reason, it is also necessary to inform consumers about good hygiene practices that should be implemented at home.

## 3. Main Foodborne AMR Microorganisms

In foodborne bacteria, the AMR is an escalating global public health challenge, threatening the effectiveness of antibiotics and food safety [49]. In the EU, the European Food Safety Authority (EFSA) and the European Center for Disease Prevention and Control (ECDC) analyze AMR data collected annually by the EU Member States and reporting countries and publish the findings in an annual EU Summary Report.

European AMR surveillance (Decision 2020/1729) focuses on key pathogens (*Salmonella*, *Campylobacter*) and commensal indicators (*E. coli*) in food-producing animals [50]. The regulatory framework requires specific testing for strains resistant to critical antibiotics, such as beta-lactamase (ESBL)-, AmpC, and carbapenemase (CP)-, while at the national level, monitoring is often extended to *Staphylococcus aureus* (MRSA) in food and livestock [51].

High-resistance frequencies were documented across major bacterial groups: Gram-negative isolates were predominantly resistant to aminoglycosides, cephalosporins, fluoroquinolones, penicillins (PEN), sulfonamides, and tetracycline. Conversely, Gram-positive bacteria primarily exhibited resistance to glycopeptides, lincosamides, macrolides, and nitrofurans [52]. These pathogenic zoonotic microorganisms are disseminated into the surrounding environment through both airborne organic dust and surface-contaminated waters, causing human infections and also acting as environmental reservoirs of ARGs [7,53].

### 3.1. Enterobacterales

Enterobacterales play a critical role in the spread of AMR in the food chain. Recently, MDR Enterobacterales, which were confined to the hospital environments, are now emerging in the domestic food-related environments as well [45]. Species like *E. coli*, *Salmonella* spp., and *Klebsiella* spp. can not only act as reservoirs for resistance genes but can also transfer them to pathogenic and commensal microorganisms, amplifying the risk throughout all stages of food production, from primary production to consumption [54]. Resistance in commensal *E. coli* strains is used as a general indicator of the selective pressure of antibiotics in zootechnical environments [51]. As reported by the EFSA [51], between 2021 and 2023, carbapenemase (CP)-producing *E. coli* strains were detected in samples from broilers, fattening turkeys, fattening pigs, and cattle under 1 year of age. The most commonly identified CP genes included *bla*_OXA−48_, *bla*_OXA−181_, *bla*_OXA−244_, *bla*_NDM−5_, and *bla*_VIM−1_.

In a study conducted by Azevedo et al. [45], antimicrobial susceptibility was assessed in 125 Enterobacterales isolates obtained from domestic food-related environments. Nearly half of the isolates (49.6%) showed resistance to at least one antimicrobial agent, with ampicillin resistance being the most frequent (32.8%). Lower resistance levels were reported for nitrofurantoin (6.4%), tetracycline (4%), nalidixic acid (3.2%), chloramphenicol (2.4%), and trimethoprim (1.7%), while multidrug resistance occurred in 6.4% of the strains. It should be noted that this problem is not limited to some kinds of food, covering many different types.

The study by Al-Kharousi et al. [40] on Enterobacterales isolated from fresh fruits and vegetables showed that 74% of the isolates exhibited resistance to at least one antibiotic, while 16% were classified as multidrug-resistant. The highest AR showed by Enterobacteria isolates was against antibiotics like ampicillin (66%) and cephalothin (57%), followed by amoxicillin–clavulanic acid (33%), cefoxitin (31%), tetracycline (9%), nalidixic acid (7%), trimethoprim (6%), and kanamycin (5%). In addition, three isolates showed intermediate resistance to imipenem. *E. coli* isolated from lettuce exhibited MDR against five antibiotics, and 15 isolates were confirmed to have AmpC β-lactamase.

It should be noted that Enterobacteria are known for their ability to produce biofilms, which represent an important hot spot for horizontal gene transfer (HGT) within and between bacterial species. Laconi et al. evaluated the biofilm-producing ability of ESBL/pAmpC-producing *E. coli* strains (isolated from the broiler production pyramid), reporting not only the high biofilm-producing capacity of these strains but also the possible association between resistance genes (such as *bla*_CTX-M-type_) and an increased biofilm production [55].

#### 3.1.1. *E. coli*

Given the high ubiquity of *E. coli*, this microorganism often serves as a primary indicator organism for AMR. It is considered more representative of the overall resistance landscape and reservoir of transmissible genes in food-producing animals than other less common zoonotic bacterial species. At the EU level, between the four animal populations monitored, no differences in the low-level resistance to cefotaxime, ceftazidime, azithromycin, and colistin were observed. In Italy, in 2022, meropenem resistance was reported in one of *E. coli* isolates from turkeys.

Resistance to carbapenem is still uncommon in commensal *E. coli* from food-producing animals in Europe. These findings regarding the resistance of cefotaxime and ceftazidime (third-generation cephalosporins) and carbapenem relate to commensal *E. coli* isolates recovered using non-selective culture methods. The median levels of resistance to ciprofloxacin and nalidixic acid among *E. coli* isolates from young pigs and cattle were low at the EU level.

Conversely, the median levels of ciprofloxacin and nalidixic acid resistance were very high in broilers and in turkeys. Considering all reporting MSs, the median level of resistance to colistin was rare. Higher levels of resistance were registered in broilers in Cyprus and turkeys in Poland and Portugal. Decreasing trends in the levels of colistin resistance were reported among isolates from specific animal populations, due to the fact that sales of polymyxins, such as colistin, for use in animals, in Europe, between 2017 and 2022, have decreased by over 40% [56]. The resistance to ampicillin, sulfamethoxazole, trimethoprim, and tetracyclines was generally common at the MS-group level, in indicator *E. coli* for all animal populations, apart from trimethoprim resistance in isolates from young cattle and turkeys. The frequent occurrence of resistance to these substances reflects the widespread past and present use of these antimicrobials in food-producing animals.

#### 3.1.2. *Salmonella* spp.

*Salmonella* is one of the most frequently isolated foodborne pathogens and currently represents one of the main public health issues in the world. As reported by EFSA, the number of human isolates of *Salmonella* spp. reported tends to vary considerably between EU/EEA countries [51]. A large number of these human isolates showed high resistance to ampicillin, sulfonamides, and tetracyclines, while resistance was moderate to very high in isolates from farm animals and imported poultry meat, with the exception of laying hens, for which low resistance was reported.

Between 2013 and 2022, the decrease in resistance in *S*. Typhimurium, commonly associated with young cattle and pigs under 1 year of age, led to a significant decrease in resistance to ampicillin and tetracyclines in isolates from humans. In 2022, a very high overall resistance to fluoroquinolones (ciprofloxacin) was observed among isolates from chickens and turkeys, at 55.5% and 57.9%, respectively. This level of resistance was high in laying hens (24.7%). In the same year, the overall resistance of human isolates of *Salmonella* to ciprofloxacin was 18.7%, with the lowest levels observed in monophasic *S.* Typhimurium (9.6%) and high to extremely high levels in *S.* Infantis (40.1%) and *S.* Kentucky (72.7%). In 2021, moderate levels of resistance were observed in *Salmonella* isolates from farm including cattle under 1 year of age (12.7%) and fattening pigs (10.1%). Extremely high ciprofloxacin resistance was observed in isolates of *S*. Kentucky from fattening turkeys (100%), broilers (84.2%), and laying hens (82.1%). Conversely, meropenem resistance remained rare in 2022 (<0.1%). While combined resistance to fluoroquinolones and third-generation cephalosporins was generally negligible (1.0%) across human and animal isolates, certain serovars showed concerning exceptions: *S*. Kentucky from broilers and *S*. Infantis from turkeys reached high (21.1%) and moderate (16.9%) levels, respectively. In humans, resistance to these serovars was moderate in *S*. Kentucky (12.2%) and low in *S*. Infantis (5.9%). Although carbapenemase-producing (CP) *Salmonella* was absent in animal populations during 2022–2023, human clinical cases were identified (5 in 2022; 6 in 2023), predominantly involving *bla*_OXA-48_ or *bla*_OXA-48_-like genes.

Considering this, the increasing spread of AR strains of *Salmonella* may increase the risk of infection in the population, potentially leading to higher hospitalization rates, less effective treatments, longer hospital stays, and higher healthcare costs. In light of this, it would be advisable to adopt preventive measures and policies to address this issue, increasing the surveillance activities mainly in intensive farming contexts.

#### 3.1.3. *Klebsiella* spp.

The *Klebsiella* species, particularly *Klebsiella pneumoniae*, poses a growing risk to public health due to its ability to act as an opportunistic pathogen and its high AMR within the food chain. *Klebsiella* spp. is a ubiquitous bacterium found in soil, water, and the gastrointestinal tracts of humans and animals. Particularly in the latter, the use of antibiotics for non-therapeutic purposes promotes the emergence of AMR *Klebsiella* strains [57]. *K. pneumoniae* is a common contaminant found in various types of food products, particularly raw foods.

A study found a *K. pneumoniae* prevalence of 38% in raw food samples, and the raw milk samples were predominantly contaminated (19/51), followed by fruits (12/51), meat (11/51), and vegetables (9/51) [57]. In particular, among vegetable products, in the same study, parsley and lettuce showed the highest contamination rates (62.5% and 50%, respectively). Furthermore, the same researchers also reported that among the isolates, 43% produced ESBL, 24% produced AmpC, and 20% produced carbapenemase.

Abebe and colleagues analyzed the prevalence, biofilm formation capacity, and AMR of *K. oxytoca* and *K. pneumoniae* strains isolated from cheese and minced meat [58]. Resistance patterns differed slightly between species. In *K. oxytoca* (n = 19), the highest resistance rates were reported for streptomycin and kanamycin (73.7% each), followed by ampicillin (63.2%). In *K. pneumoniae* (n = 5), most isolates were resistant to kanamycin and streptomycin (80% each), while amikacin resistance was observed in 60% of the strains. Generally, the majority of *K. oxytoca* and *K. pneumoniae* isolates showed strong biofilm production under different growth conditions, and the majority of the isolates were also resistant to antibiotics.

As reported by ECDC and WHO, resistance to key antibiotics, specifically third-generation cephalosporins and carbapenems, was more prevalent in *K. pneumoniae* than in *E. coli*. In 33% of reporting nations, the resistance frequency for *K. pneumoniae* reached or surpassed 25% [59]. In Spain, fresh vegetables were found to be contaminated with MDR strains of *K. pneumoniae*, and the presence of *K. variicola*-producing OXA-181 carbapenemase has been previously documented in fresh vegetables imported into Switzerland [60,61]. The spread of AMR in *K. pneumoniae* shows marked geographical variability in Europe. In particular, resistance rates tend to be higher in south-eastern European countries than in the north-western parts. In 2021, resistance percentages to third-generation cephalosporins (cefotaxime/ceftriaxone/ceftazidime) below 10% were observed in Austria, Denmark, Finland, Iceland, Norway, Sweden, and Switzerland, while 19 of 45 countries reporting data on this microorganism, particularly in the southern and eastern parts of the Region, reported AMR percentages of 50% or above. This geographical difference also emerged considering *K. pneumoniae* resistance to carbapenems (imipenem/meropenem), with 14 out of 45 countries reporting AMR rates below 1%; 15 countries reported rates equal to or above 25%, and 8 countries reported AMR rates equal to or above 50% [59]. Differences in the reported percentages of AMR *Klebsiella* spp. across the food chain are likely driven by several contextual determinants. Specifically, distinct antibiotic consumption patterns in human and veterinary medicine exert selective pressure, favoring the persistence of resistant strains. Furthermore, the surveillance systems and intensity may influence the detection of AMR *Klebsiella* spp., thereby complicating the comparability of international datasets [62].

It should be noted that sales outlets may also influence the risk of food contamination by *Klebsiella* spp. Junaid et al. found that products purchased from street vendors had higher contamination rates (45.1%) than those purchased from local markets (29.4%) and supermarkets (25.5%), probably due to less hygienic practices [57].

### 3.2. Campylobacter spp.

In 2023, AMR surveillance in human-isolated *C. jejuni* and *C. coli* involved 24 Member States (MSs) and 2 non-MS (Iceland and Norway). During the same period, AMR on the same bacterial species was analyzed in cattle under 1 year from 11 MSs and 1 non-MS, and in fattening pigs from 27 MSs and three non-MSs. The resistance rates showed marked variability between reporting countries, between antimicrobials, and between the two *Campylobacter* species.

In food-producing animals, the highest levels of resistance to ciprofloxacin were reported in *C. coli* isolates, with rates ranging from 54.3% (fattening pigs) to 84.1% (fattening turkeys). *C. jejuni* isolates from poltry in 2022 showed an extremely high resistance to ciprofloxacin (78.2% in fattening turkeys and 70.9% in broilers). Differently, combined resistance to ciprofloxacin and erythromycin, both critically important antimicrobials used against campylobacteriosis, was generally rare to low in *C. jejuni*, both in humans and food-producing animals. However, for *C. coli*, the combined resistance was low in humans (7.1%), broilers (8.2%), and fattening pigs (9.1%) isolated strains; moderate in fattening turkeys (17.4%), and high in cattle under 1 year of age (32.7%), representing a potential risk for public health. In general, complete susceptibility (CS), defined as susceptibility to ciprofloxacin, erythromycin, tetracycline, and gentamicin, was higher in *C. jejuni* compared to *C. coli* isolates. In 2023, the CS from *C. jejuni* in humans was 25.5%, while in food-production animals, it ranged from 16.5% (fattening turkeys) to 51.1% (fattening pigs).

MDR, defined as resistance to three or more antimicrobials among ciprofloxacin, erythromycin, tetracycline, and gentamicin, was generally very low (0.7%) for human isolated *C. jejuni* and ranged from very low to low in the animal species considered. Differently, MDR was markedly higher in *C. coli*, specifically occurring in 8.6%, 34.8%, 16.9%, 10.7%, and 8.3% of isolates from human, cattle under 1 year of age, fattening turkeys, fattening pigs, and broilers, respectively. These results agree with the higher levels of resistance to selected antimicrobials seen in *C. coli* isolates.

From 2014 to 2023, human *C. jejuni* resistance to ciprofloxacin increased in 11 MSs and decreased in 3 reporting countries (2 MSs and 1 non-MS). Resistance to ciprofloxacin in *C. jejuni* showed differing trends, increasing in 6 MS for broiler chickens and in one for fattening turkeys, while decreasing in 3 and 1 MS, respectively. Human *C. coli* isolates showed an increase in ciprofloxacin resistance in two MS and a decrease in two others. In contrast, erythromycin resistance showed a predominantly declining trend: it decreased in human *C. jejuni* in ten countries (9 MS and 1 third country), as well as in isolates from broiler chickens (6 MS) and turkeys (2 MS). Similarly, erythromycin resistance in *C. coli* decreased in 9 MSs for human cases and in 4 MSs for fattening pigs. The rare upward trends for erythromycin were limited to *C. jejuni* in three cases (two human, one meat) and to a single MS for human *C. coli* [51].

### 3.3. Listeria monocytogenes

Agricultural environments, often characterized by poor hygiene, inadequate manure management, and contaminated resources serve as important reservoirs for *L. monocytogenes*, facilitating its introduction into the food chain [63,64]. Within processing facilities, the pathogen’s ability to form resistant biofilms on equipment ensures its persistence despite sanitization, particularly in the cold and humid conditions typical of ready-to-eat (RTE) food production lines [65,66]. This environmental resilience poses a serious risk to consumer safety [67]. In addition, chronic exposure to sublethal concentrations of disinfectants and antibiotics can select for multidrug-resistant strains that often harbor co-localized virulence genes [68,69]. As a result, consumption of contaminated RTE meat, dairy, and seafood remains a major cause of human listeriosis.

Frequent resistance in *L. monocytogenes* to different antibiotic classes—including penicillins (e.g., penicillin G and ampicillin), quinolones (e.g., norfloxacin), aminoglycosides, lincosamides, macrolides, tetracyclines, trimethoprim—poses a significant challenge to the clinical management of listeriosis [70,71].

### 3.4. Methicillin-Resistant Staphylococcus aureus (MRSA)

MRSA, a ubiquitous microorganism found on the skin and mucous membranes of humans and animals, is one of the main causes of infection in hospital environments. Based on epidemiological features, it can be divided into three broad categories: community-associated (CA-) MRSA, healthcare-associated (HA-) MRSA, and livestock-associated (LA-) MRSA. HA-MRSA and CA-MRSA are mainly related to human infections, while LA-MRSA has been detected in most farm-animal species, including those covered by the AMR monitoring according to Decision EU/2020/1729.

In accordance with EFSA technical guidelines, the Commission Implementing Decision EU/2023/1017 updated the previous regulation (EU/2020/1729) by introducing mandatory MRSA monitoring in fattening pigs for 2025 [72,73]. The aim is to estimate the spread of MRSA at the European level by testing healthy animals directly at the slaughterhouse. It should be noted that, in the two-year period 2022–2023, only a small number of Member States have submitted data on the presence of MRSA in food.

Between 2022 and 2023, turkey meat recorded the highest rate of MRSA contamination. In particular, Germany detected the bacterium in 53.7% of carcasses and 34.3% of retail meat (2022), while the Netherlands reported positivity rates of 50.0% and 28.6% in 2022 and 2023, respectively.

For broiler chickens, retail prevalence ranged from 1.1% (Spain) to 9.7% (Netherlands). In pigs, the highest rates in 2023 were reported by Slovakia (15.4%) and Austria (14.4%). Finally, in cattle, the Netherlands found a higher presence in processing plants than in retail, while Germany detected MRSA in 6.8% of imported beef.

Between 2022 and 2023, turkey meat recorded the highest rate of MRSA contamination. In particular, Germany detected MRSA in 53.7% of carcasses and 34.3% of retail meat (2022), while the Netherlands reported positivity rates of 50.0% and 28.6% in retail meat in 2022 and 2023, respectively.

In retail meat from broiler, retail prevalence ranged from 1.1% (Spain) to 9.7% (Netherlands). A slight increase from 7.5% in 2022 to 9.7% in 2023 was reported by the Netherlands. In 2022, Germany reported a MRSA contamination in 4.9% of retail meat from broilers, while 16.7% of broiler carcasses sampled at slaughterhouses were MRSA positive. The Netherlands reported an MRSA occurrence of 6.9% in 2023 and 7.2% in 2022, in retail meat from pigs. Germany reported MRSA in 10.3% of retail meat from pigs, while the highest occurrences were reported by Austria (14.4%) and Slovakia (15.4%) in 2023. Data from the Netherlands show a higher prevalence of MRSA in processing plants (9.1% in 2022 and 13.6% in 2023) than in samples taken at retail meat from cattle (6.1% and 3.9%). In the same sector, Austria recorded a positivity rate of 1.9% in retail sales, while Germany detected MRSA positivity in 6.8% of beef imported at border control posts. Finally, minor reports concerned duck meat in Germany (1.9% in retail and 0.9% in carcasses in 2022) and sheep meat in the Netherlands (4.6% in retail in 2023). Germany also reported MRSA in retail meat from duck (1.9%) and duck carcasses sampled at a slaughterhouse (0.9%) in 2022, while the Netherlands reported MRSA in 4.6% of retail meat from sheep sampled in 2023 [51].

### 3.5. The Potential Role of Probiotic Microorganisms as Carriers of ARGs

FAO and WHO defined probiotics as “Live microorganisms which when administered in adequate amounts confer a health benefit on the host” [74]. As is well known, the intake of probiotics and fermented foods rich in probiotics is strongly recommended by the international scientific community, given the significant positive effects for human health. However, in recent years, several studies highlighted that different probiotic microorganisms may contain ARGs [75,76,77,78,79]. The human gut, with its extremely high bacterial density and constant selective pressures (derived from diet, drugs, and other factors), is a “hotspot” for the vertical transfer of genes from probiotic bacteria to commensals and pathogens [75,76].

Probiotic bacteria, like *Lactobacillus* and *Bifidobacterium*, can act as reservoirs of ARGs [77]. Since these microorganisms are deliberately introduced into the food chain, the presence of transferable ARGs poses a serious risk for public health. Shotgun metagenomic analysis performed on probiotic products identified over 70 different types of ARGs, including mutant *rpoB* genes (rifampicin resistance), *tet(W/N/W)* genes (tetracycline resistance), and potentially *TEM-116*-encoding ESBLs [80]. In another study, 30 probiotic strains (including *L. rhamnosus*, *L. acidophilus*, *L. casei*, *L. reuteri*, *L. plantarum*, and *L. fermentum*) isolated from 19 commercial products demonstrated a high level of resistance toward nalidixic acid, vancomycin, kanamycin, teicoplanin, co-trimoxazole, amikacin, streptomycin, norfloxacin, cefepime, and nitrofurantoin [76]. It should be noted that the presence of MGEs increases the risk of ARGs transmission from one species to another. The *tet(W/N/W)* gene was found to be associated with the ISBian1 insertion sequence in strains of *Bifidobacterium animalis* [80]. Rozman et al. reported that the prevalence of the *tetW* gene was higher in analyzed bifidobacteria (31.9%) than lactobacilli (6.3%) [77]. Genomic analyses performed by Fatahi–Bafghi and colleagues on a group of bacteria with probiotic properties revealed that a high proportion of the ARGs identified, estimated at between 87% and 90%, are associated with MGE [81]. This evidence indicates that most of the ARGs detected in probiotics have a high intrinsic potential for horizontal transfer.

In light of the available scientific evidence, the safety protocols of the entire probiotics industry need to be updated, requiring companies to perform antibiotic susceptibility testing on a broad panel of molecules before using a microorganism as a probiotic.

It should be noted that this transmission of ARGs can also occur with pathobiont microorganisms. Pathobionts are members of the resident microbiota that generally coexist with the host without causing disease but may become opportunistic pathogens under specific conditions, such as immune imbalance or microbial dysbiosis [82]. They can act as reservoirs of ARGs and contribute to their dissemination through horizontal gene transfer within microbial communities, and the most common pathobiont strains are *Proteobacteria* species *E. coli* and *P. mirabilis*, and *Enterococcus faecalis* [83]. Rowan–Nash et al. reported that the genomes of pathobionts harbor a higher proportion of ARGs than those of typical intestinal commensals such as *Bacteroides* and *Bifidobacterium* [84].

These findings indicate that, although the commensal microbiota is recognized as a reservoir of AMR, the expansion of pathobiont populations may play a key role in increasing the abundance of ARGs within the gut microbiome.

## 4. Molecular Mechanisms for AMR in Foodborne Bacterial Species

The development of molecular mechanisms for AMR takes advantage of the adaptability of microbial genomes. Main resistance patterns that can be displayed are based on reduced drug binding and/or entry into the cell; prompt drug efflux; increased enzymatic drug cleaving; activation of alternative metabolic ways; genetic modification of drug targets [85]. A recent review [86] highlighted that almost 150,000 genomes from AMR foodborne pathogens isolated from the food chain can be retrieved from the NCBI Pathogen Detection database. The same authors calculated that most of the genomes from food sources were *Salmonella* (47.88%), *Campylobacter* (23.03%), *Escherichia* (11.79%), and *Listeria* (11.3%). This huge number offers a high amount of information about resistance gene spread and resistance molecular mechanisms development in the food chain. Just to give a few examples, *Salmonella* isolates from meat/poultry are found to harbor ARGs associated with multiple resistance classes such as tetracycline, β-lactams, quinolones, and sulphonamides, while *B. cereus* from meat/poultry and fruit/vegetable sources are more likely to carry tetracycline resistance and/or the *van*R-A/*van*S-Pt glycopeptide resistance cassette(s), and MRSA strains encoding the *mec* cassette were isolated from the entire pork chain [87].

The AcrAB-TolC system in *E. coli* and *Salmonella*, the CmeABC system in *Campylobacter*, and the EmrAB and MdfA in *Salmonella* are typical efflux pumps that reduce the intracellular concentration of tetracyclines, macrolides, and fluoroquinolones through active transportation [88]. A classic example of drug enzymatic breakdown is instead given by the β-lactamases, able to hydrolyze β-lactams, like the ESBL in *Salmonella* encoded by *bla*TEM or the *bla*CTX family [89]. Modifications of target site in the quinolone resistance-determining regions (QRDRs) of *gyr*A and *par*C are also present in *Salmonella* and *Campylobacter* resistant to fluoroquinolones [90], while methylation of 23S rRNA, contributes to macrolide resistance.

Colistin resistance relies on the lipid A modification by a phosphoethanolamine transferase encoded by the *mcr* genes, able to reduce drug binding [91].

These features may be intrinsic, acquired, or adaptive.

### 4.1. Intrinsic Resistance

Intrinsic resistance refers to microbial natural capacity to resist certain antibiotic classes. This property is owned by bacteria thanks to their already present chromosomal genes without further mutations or gain of external genes. Thus, it is independent of antibiotic selective pressure and horizontal gene transfer (HGT). Inherent resistance models rely on lack of affinity between the drug and the bacterial target; inaccessibility of the drug into the bacterial cell; extrusion of the drug by chromosomally encoded efflux pumps; and presence of drug-degrading enzymes.

Intrinsic resistance to vancomycin naturally occurs in *E. coli* due to its structural and physiological traits, such as reduced membrane permeability and effective efflux [92]. This kind of inherent resistance to glycopeptide antibiotics is present in Gram-negative species, due to their outer membrane. *Campylobacter* species are resistant to multiple antibiotics, like penicillin, cephalosporins, and vancomycin [93]. Natural resistance to antibiotics targeting the cell wall and associated functions (β-lactams, glycopeptides) is present in mycoplasma, due to the lack of a cell wall.

The consequences of intrinsic resistance are that these bacteria will be unaffected by certain antibiotic treatments when used in clinical applications.

### 4.2. Acquired Resistance

Acquired resistance is essentially an evolutionary process by which a previously sensitive bacterium becomes resistant to one or more antimicrobials through the acquisition of chromosomal gene mutations or gaining exogenous genetic material. A few cases of acquired resistance depend on mutations in the bacterial chromosome, which take place in genes encoding for drug targets, transporter proteins, regulatory proteins, and antibiotic-modifying enzymes [85,94]. Mutation is a spontaneous event that takes place regardless of the presence of the antibiotic. The mutated bacterial cell developed an advantage with respect to the susceptible cells, which are killed by the antibiotic, leaving a resistant subpopulation.

The second condition occurs by means of HGT. Indeed, MGEs can carry resistance genes and transfer these genes across various bacterial strains, both commensal and pathogenic. By the way of MGE, the AMR spread easily occurs, leading also to MDR features [95].

#### Horizontal Gene Transfer

The genetic material responsible for resistance features can horizontally pass across bacterial cells through different processes. Transformation, transduction, and conjugation are the basic transmission routes for the acquisition of ARGs.

MGE involved with the dissemination of AMR are most commonly plasmids (conjugative and mobilizable), but also other elements, like integrons, gene cassettes, transposons (insertion sequences, IS; conjugative transposons) [96,97].

Transferable resistance was first identified decades ago, when resistance genes carried by *Shigella* were passed to *E. coli* via plasmids. Plasmids can carry and transfer multiple resistance genes, like the β-lactamase (*bla*) or the *mcr* gene families. ARG dissemination can occur intra- or interspecies: conjugative plasmids are able to transfer ARGs across bacterial genera, even if phylogenetically distant [85]. The pESI-like plasmid recently described in *S.* Infantis has a main role in spreading multiple resistance genes [98].

Transposons are called “jumping genes”, since they can move into different places of the genome: they can transfer from a plasmid to other plasmids or from a DNA chromosome to plasmid and vice versa, thus causing the spread of ARG [99]. The *mec*A gene of MRSA is supposed to have been acquired by transposition [100,101].

Class 1 integrons are commonly identified in *Salmonella* and *E. coli* from animal and food sources, often linked to MDR [102].

ISs are a subset of DNA transposons that can determine AMR not only through ARG transfer but also causing genes inactivation in the insertion site by direct integration [99]. An example of IS is the IS256, responsible for resistance to aminoglycosides [99].

Transposons can also be transmitted among bacterial species by bacteriophages, as frequently happens in staphylococci. The transduction is the mechanism that allows the propagation of DNA sequences through bacteriophages.

Lastly, the transformation occurs when free genetic material, often released from a dead bacterial cell, is taken up by other competent bacteria. This mode is increasingly highlighted as an important route in the environment [103].

HGT across the pork production chain, from swine farm, slaughterhouse, and retail market, has been recently reported by Yang et al. [104].

### 4.3. Adaptive Resistance

Adaptive resistance is a phenotype adaptation to environmental changes, often developed after exposure to subinhibitory concentrations of antibiotics along with other environmental stresses (nutrition, pH, growth factors, etc.). In the majority of cases, adaptive resistance is transient and reverts back after inducing conditions are removed. Clarification of the mechanism of adaptive resistance and its interplay (mutations, gene amplification, efflux pumps, biofilm formation, epigenetics, heterogeneity, and colonial attributes) is not well understood and deserves further studies [105,106].

However, the role of the microenvironment has been characterized in the biofilm, where AMR is promoted by the particular physiological state. Indeed, changes in gene expression, increase in HGT thanks to bacterial proximity, and decrease in metabolic activity, all contribute to the expression of a tolerant phenotype [95]

Bacterial biofilms developed in food processing environments (FPEs) constitute a microenvironment in which AMR propagation is fostered. Additionally, in FPEs a further selective pressure for AMR is promoted by the use of disinfectants and/or the presence of environmental contaminants. Indeed, the close presence of genetic determinants of disinfectants and/or heavy metals and antibiotic resistance, both present in MGE [107,108] determine their co-selection and simultaneous spread.

### 4.4. Environmental Resistome

The resistome is defined as the complete set of ARGs associated with a microbial community of both pathogenic and non-pathogenic bacteria, present in food and associated environments. Particularly, besides bacterial species traditionally associated with food products, like commensals and zoonotic agents, environmental microorganisms present in food processing plants can act as carries and spreaders of AMR genes [109].

Resistome characterization is of paramount importance in understanding AMR epidemiology and spread, especially in the food chain [110]. Nevertheless, despite the majority of studies focused on specific pathogen-resistance associations [111,112], the diversity of resistant bacteria and ARGs in food production systems remains poorly characterized. A very recent investigation by Quijada et al. [109] underlined that prevention of AMR spread among the food chain would benefit from a better comprehension of ARG diversity in bacteria circulating through FPE of different sectors and in end food products.

The metagenomics analysis is the investigation tool that may provide the best characterization of bacterial community composition and related resistome across multiple reservoirs in the food chain, instead isolating and separately analyzing single species. Complete resistome information in a certain environment through metagenomics whole sequencing allows us to skip the culture of single isolates, which could be time-consuming and not completely representative of the real situation, if sometimes the microorganisms do not grow due to the stress (but not kill) given by the use of detergents or disinfectants at sublethal doses or other food treatments. To this end, molecular surveillance by NGS of ARGs worldwide, from the primary production to the FPE (slaughter, transformation, processing, packaging, transportation), allows in-depth monitoring of AMR spread and evolution. However, some limitations have also been attributed to metagenomics analysis, among which the detection of silent ARGs (i.e., genes whose presence is not associated with a corresponding resistant phenotype), leading to the lack of phenotypic confirmation, should be mentioned.

A recent comprehensive investigation [109] analyzed a huge number of raw-materials, end-products and surface samples from many food processing facilities, highlighting that the majority of known ARGs (>70%), with higher presence of antibiotic classes most widely consumed in the EU (tetracyclines, β-lactams, aminoglycosides and macrolides), including many conferring resistance to critically important antimicrobial WHO list [113], circulates daily throughout various food production chains.

The same study obtained evidence of sector-specific resistomes, especially in the meat and dairy sectors, and the highest ARGs load and diversity was recovered from production contact surfaces, which are principal sources of ARGs, mainly carried by MGE. Interestingly, the greatest contribution to the food resistome was found in commensal bacteria and also in some microorganisms exploited for their useful properties in food production.

Therefore, since the AMR pattern carried by food items is highly influenced by the production environment and the ecological pressure determined by processing, a constant monitoring of food processing environment resistomes in an internationally coordinated system is recommended.

Understanding the molecular mechanisms underlying antimicrobial resistance is essential for the development of effective mitigation strategies. Insights into mechanisms such as horizontal gene transfer, efflux pumps, and biofilm formation can inform targeted interventions, including improved surveillance of resistance genes, the development of novel antimicrobial agents or inhibitors, and the implementation of more effective sanitation and stewardship practices across the food chain.

## 5. Public Health Implications and Integrated Strategies for Mitigation and Control

The development of antimicrobial resistance and the emergence of multi-drug-resistant (MDR), pan-drug-resistant (PDR), and extensively drug-resistant (XDR) pathogens, carrying novel resistance mechanisms, represent a growing global threat to both human and animal health [114].

AMR has been defined by the World Health Organization as one of the priority challenges for health systems [1,2]. Both nosocomial and community-acquired infections caused by AMR microorganisms are associated with a marked reduction in the effectiveness of standard treatment regimens, resulting in an increased risk of progression to severe disease, treatment failure, complications, and mortality [115,116]. From 1990 to 2021, deaths from AMR increased by over 80% for adults 70 years and older [1].

The ECDC estimates that in the European Union alone, antimicrobial resistance causes 33,000 deaths and approximately 880,000 cases of disability each year [117]. In addition to the clinical burden, this leads to substantial increases in healthcare costs and increased pressure on infection surveillance and control systems.

Alarming levels of AMR have been reported in all countries, regardless of their income level.

In an increasingly interconnected global context, antimicrobial resistance can disseminate rapidly among humans, animals, plants, and the environment, including soil, surface water and groundwater [118,119].

Several studies have highlighted a close genetic correlation and overlapping antibiotic resistance profiles between AMR microorganisms isolated from food/animals/environment and humans, suggesting possible transmission dynamics between different reservoirs [120,121,122].

As a result, strategies limited to a single sector are inadequate to effectively prevent and control AMR [123]. Without harmonized action on a global scale, the world risks entering a post-antibiotic era in which common infections could once again become lethal.

To address this emergency, the WHO in 2015 promoted the Global Action Plan on antimicrobial resistance [124], approved by the 68th World Health Assembly [125], in which the Assembly members recognized that rising antimicrobial resistance seriously threatens the future management of current and emerging pathogens.

According to the Global Action Plan, a problem of such complexity needs to be addressed with coordinated multi-sectoral interventions proper to the “One Health” approach, integrating the human, veterinary, and environmental sectors.

The One Health approach, therefore, involves bringing together relevant stakeholders to communicate and work jointly on the development, implementation, and monitoring of programs, policies, legislation, and research aimed at mitigating antimicrobial resistance. Therefore, strong coordination among the various stakeholders is essential, including researchers in the field of infectious diseases and microbiology; funders of research and new drug development; companies engaged in antibacterial drug development; health care professionals; veterinarians; and national and global policy makers [117,126].

The World Health Assembly also urged all Member States to develop and implement national action plans (NAPs) on antimicrobial resistance by 2017, in line with the objectives of the global action plan. As of November 2023, 178 countries have developed AMR National Action Plans for guiding national strategy and action for AMR response [127,128]. The NAPs include policies promoting the prudent use of antibiotics, surveillance and monitoring systems, education and training initiatives, research activities, and other measures.

In particular, antimicrobial stewardship is central among the various actions to be implemented. Efforts to promote the conscious and appropriate use of antibiotics need to be stepped up, with targeted campaigns aimed at healthcare workers, veterinary professionals, livestock farmers, and the general population. A large proportion of antimicrobials are used in animal production [129], so in veterinary medicine, it will be necessary to raise the level of knowledge, training, and awareness among veterinarians regarding the problem of AMR.

## 6. Gaps in the Global AMR Response and the Need for Strengthened Actions

Despite the recommendations and global strategies promoted by the World Health Organization and other international bodies, and despite the implementation of specific AMR National Action Plans in many countries, antimicrobial resistance continues to be one of the main threats at a global level.

Among the main challenges and difficulties in managing AMR in humans, animals, and the environment are the ubiquitous spread of AMR microorganisms and resistance genes, their rapid cross-border transmission due to globalization, the high adaptive capacity of these microorganisms, the limitations of currently available diagnostic tests, gaps in surveillance systems, and the limited availability of new effective treatment options against priority pathogens.

The improper use of antibiotics in various contexts, especially in veterinary medicine, continues to favor the constant spread of resistant strains. At the same time, fragmented or insufficiently timely monitoring systems reduce the ability to detect new patterns of resistance early and implement effective control measures.

Furthermore, effective integration between the human, veterinary, and environmental sectors, as envisaged by the One Health approach, is still limited. In this regard, the Third Global High-Level Ministerial Conference on Antimicrobial Resistance, held in Oman in November 2022, highlighted the urgent need for increased support from governments and philanthropic organizations for research initiatives addressing AMR within a One Health framework. Although there are multiple research programs and initiatives underway, the degree to which these efforts effectively address AMR across the human, animal, environment interface remains limited.

To date, only a small proportion of AMR research funding (approximately 6%) has been directed toward projects adopting a truly multisectoral approach [123]. Furthermore, investments in research and development, prevention, and control remain insufficient.

Further actions will be required to strengthen strategies to combat AMR, including the improvement of microbiological diagnostics, particularly through the implementation of rapid and molecular tests, in order to enable timely and targeted therapies and to reduce the use of inappropriate empirical treatments. In parallel, the development of new therapeutic strategies, including innovative molecules, pharmacological combinations, and alternative approaches, is crucial.

Since 2017, 13 new antibiotics targeting bacterial priority pathogens have received regulatory approval, with several of these agents being included in the WHO Essential Medicines List. Nevertheless, despite these advances, antimicrobial resistance continues to progress, with increasingly complex resistance profiles emerging, including reduced susceptibility to many recently introduced antibiotics [130].

Given the high quantity of antibiotics used in animal production, effective interventions of antimicrobial stewardship in the veterinary and zootechnical fields are a key lever for reducing overall selective pressure and combating the spread of AMR. In this context, priority should be given to strengthening good farming practices and reducing intensive farming, improving hygiene standards, strengthening biosecurity measures, optimizing animal welfare, and implementing antimicrobial stewardship programs. These programs should be based on core principles that include [117]:

(a) Prudent and justified use of antimicrobials: antimicrobials should be administered only when clearly indicated, avoiding routine or prophylactic use. Treatment decisions should be made under veterinary supervision and based on a careful assessment of clinical necessity.
(b) Appropriate selection of antimicrobials: when antimicrobial therapy is necessary, the choice should be guided by appropriate diagnostic tests and, where possible, antimicrobial susceptibility testing, to ensure targeted and effective treatment.
(c) Optimization of dosage and treatment duration: defining the correct dose and duration of therapy is essential to achieve therapeutic success while minimizing the risk of resistance selection.

Antimicrobial stewardship in veterinary medicine can be successfully implemented if veterinarians have a high level of knowledge, training, and awareness. This enables them to prescribe appropriate antimicrobials and guide farmers toward prudent use.

Furthermore, it is also important to strengthen and extend the surveillance systems for infections by AMR microorganisms, to guide choices and evaluate the impact of interventions. Effective surveillance allows for the timely implementation of all necessary measures to isolate and contain the spread of critical microorganisms. Equally important is the surveillance of antibiotic consumption, both in humans and in animals.

Surveillance and monitoring systems could be significantly improved through the adoption of the recent technological advances, such as artificial intelligence (AI) and machine learning.

AI-based tools have emerged as powerful technologies capable of analyzing large datasets derived from clinical records, laboratory data, genomic sequencing, and epidemiological monitoring systems. These models can offer valuable insights for repurposing existing drugs, identifying new antimicrobial agents, and designing combination therapies by analyzing their molecular structures. In addition, they enable the prediction of resistance patterns, the early detection of antimicrobial resistance (AMR) outbreaks, and the strengthening of antimicrobial stewardship through clinical decision-support systems [131,132,133,134].

Finally, the integration of artificial intelligence with next-generation sequencing (NGS) and metagenomic approaches allows for improved identification of antimicrobial resistance genes and the monitoring of resistomes across human, animal, and environmental reservoirs [132].

Integrating the results from the various sectors will provide a comprehensive overview of how antimicrobial resistance develops, spreads, and can be combated.

It should not be overlooked, finally, that in addition to bacterial resistance, antifungal resistance is emerging as a significant and often under-recognized public health threat. Environmental exposure to agricultural azoles and other fungicides has been identified as a key driver in the selection of resistant strains, which may contaminate agricultural environments and food products.

Antifungal-resistant pathogenic fungi have been detected not only in clinical settings [135,136,137], but also in environmental and food matrices [138,139,140], highlighting the continuum between agricultural practices, food production systems, and human health. This highlights the importance of incorporating antifungal resistance assessment into existing AMR monitoring systems to ensure a truly comprehensive One Health response.

## 7. Conclusions

Antimicrobial resistance (AMR) along the food supply chain represents a complex and evolving public health challenge, driven by the extensive use of antimicrobials, primarily in livestock farming, but also in agriculture and aquaculture.

Food and related environments act not only as vehicles of exposure for humans, but also as active reservoirs and amplifiers of AMR. Despite regulatory advances and growing awareness, significant gaps persist in the informed use of antimicrobials, harmonized surveillance, diagnostic capacity, and effective integration across sectors.

Addressing AMR in the food supply chain, as in other sectors, requires coordinated and sustained One Health actions, combining prudent use of antibiotics, surveillance, improved hygiene and biosecurity practices, and investments in innovative diagnostics and therapeutic alternatives.

## Figures and Tables

**Table 1:** Main antibiotics and their use, in horticulture and agriculture.

Antibiotic	Antibiotic Class	Use in Agriculture/Horticulture	References
Streptomycin	Aminoglycosides	It is the most widely used antibiotic in horticulture. It is used to treat bacterial diseases, mainly fire blight (*Erwinia amylovora*) in fruit trees such as apples and pears. It is also used to treat citrus greening (huanglongbing, caused by *Candidatus liberibacter* spp.).	[31,32,33]
Oxytetracycline	Tetracyclines	It is one of the most commonly used antibiotics in agriculture. It is used against fire blight (apples and pears) and bacterial spot (*Xanthomonas campestris pv. pruni*) in peaches and nectarines. It is also registered for citrus greening and bacterial canker.	[31,32,33]
Kasugamycin	Aminoglycosides	Used for seed and ear rot in rice (*Burkholderia glumae*) and for bacterial diseases in crops such as kiwifruit and citrus. Moreover, it is widely used to manage angular leaf spot in tomatoes (*Pseudomonas syringae pv. lachrymans*). It is considered an alternative treatment for bacterial fire blight.	[32,34,35]
Oxolinic acid	Quinolones	Its spectrum of activity is primarily against Gram-negative bacteria, and it is considered an alternative to streptomycin and oxytetracycline for the treatment of bacterial diseases. Also used to treat fire blight of pear and related plants, especially in areas where *E. amylovora* is resistant to streptomycin.	[32,36]
Validamycin A	Aminoglycoside/aminocyclitol	An antifungal agent known for its wide-ranging antifungal capabilities. It is used to treat bacterial wilt (*P. solanacearum*) in tomatoes, and for the control of *R. solani* in potatoes, vegetables, strawberries, tobacco, ginger, and other crops, and damping-off diseases of cotton, rice, and sugar beet.	[33,37]

## Data Availability

No new data were created or analyzed in this study. Data sharing is not applicable.
